# Invasive pressure indices in aortic stenosis: the key role of resting flow after valve replacement

**DOI:** 10.3389/fcvm.2023.1179346

**Published:** 2023-05-22

**Authors:** Muhammad Sabbah, Thomas Engstrøm, Jacob Lønborg

**Affiliations:** The Heart Center, Rigshospitalet, Copenhagen University Hospital, Copenhagen, Denmark

**Keywords:** coronary, aortic stenosis, fractional flow reserve (FFR), resting full-cycle ratio (RFR), hyperemia, coronary flow

We read the recently published consensus document concerning the management of coronary artery disease in patients with aortic stenosis (AS) undergoing transcatheter aortic valve replacement (TAVI) (1). Important newer studies were unfortunately not included (2–4). We wish to highlight their results here, as they shed light on questions raised in the consensus document relating to invasive physiological assessment of coronary lesions. In terms of physiological indices used to assess coronary stenosis severity, the most important alteration caused by AS is an increased resting flow through the coronary artery (and by extension, across a coronary stenosis) (2–4). This is not accompanied by a change in hyperemic flow or minimal microvascular resistance—neither when AS patients are compared to controls, or to serial measurements 6 months after valve replacement (2–4). Because fractional flow reserve (FFR) is based on hyperemic flow it is affected less by the presence of AS compared with non-hyperemic indices, whose cut-off is based on resting flow. The pivotal point is that resting flow is significantly reduced by the unloading effect of TAVI, whereas total hyperemic flow shows little change (4). Thus, a resting index will overestimate stenosis severity pre-TAVI, due to baseline vasodilatation, and therefore be discordant with a measurement performed after TAVI (3). In the largest cohort to date, we found no significant changes in FFR but significant improvement in resting-full-cycle-ratio (RFR) 6 months after TAVI (3). With post-TAVI FFR as a reference, pre-TAVI FFR had a positive predictive value of 91% compared to 35% with RFR. On the other hand, pre-TAVI RFR outperformed pre-TAVI FFR in terms of identifying lesions which would remain FFR negative at follow-up (negative predictive value of 100% and 87% respectively). Accordingly, we recommend that FFR be used to guide revascularization before TAVI, and RFR (and other non-hyperemic indices) to guide deferral of revascularization. The ongoing COMIC-AS study by Minten et al. which plans to include the largest sample yet (*n* = 100) may provide further evidence (5).

The authors claim, without providing a reference, that AS acts as a tandem lesion downstream of an epicardial coronary stenosis causing crosstalk between the two. This statement is to the best of our knowledge not based on experimental data. Coronary driving pressure is naturally depleted across the arteriolar/microcirculatory domain (coronary venous pressure is close to 0 mmHg) which lies between the coronary lesion and the valvular stenosis. As such, coronary lesions cannot be expected to impact the valvular stenosis in the downstream direction. Even in the case that the authors meant the valvular stenosis acts as a tandem lesion *upstream* of the coronary stenosis, the claim may not hold. Firstly, the two stenoses are not serially connected anatomically. Secondly, the entire cardiac output is ejected into the aorta at systemic arterial pressures. Unless the patient has a severely reduced systemic diastolic arterial pressure (and therefore a reduced coronary input pressure) there is no reason to assume coronary flow is inhibited by AS. Thirdly, aortic pressure, rather than left ventricular pressure, is used as the reference for distal coronary pressure when calculating FFR as well as non-hyperemic indices.
